# Evolution of the Human Diet and Its Impact on Gut Microbiota, Immune Responses, and Brain Health

**DOI:** 10.3390/nu13010196

**Published:** 2021-01-10

**Authors:** Brigitte M. González Olmo, Michael J. Butler, Ruth M. Barrientos

**Affiliations:** 1 Division of Anatomy, College of Medicine, Ohio State University, Columbus, OH 43210, USA; Brigitte.GonzalezOlmo@osumc.edu; 2 Institute for Behavioral Medicine Research, Ohio State University, Columbus, OH 43210, USA; Michael.Butler@osumc.edu; 3 Department of Psychiatry and Behavioral Health, Ohio State University, Columbus, OH 43210, USA; 4 Department of Neuroscience, The Ohio State University, Columbus, OH 43210, USA; 5 Chronic Brain Injury Program, Discovery Themes Initiative, The Ohio State University, Columbus, OH 43210, USA

**Keywords:** human diet evolution, microbiota, gut dysbiosis, neuroinflammation, neurodegeneration, depression, aging

## Abstract

The relatively rapid shift from consuming preagricultural wild foods for thousands of years, to consuming postindustrial semi-processed and ultra-processed foods endemic of the Western world less than 200 years ago did not allow for evolutionary adaptation of the commensal microbial species that inhabit the human gastrointestinal (GI) tract, and this has significantly impacted gut health. The human gut microbiota, the diverse and dynamic population of microbes, has been demonstrated to have extensive and important interactions with the digestive, immune, and nervous systems. Western diet-induced dysbiosis of the gut microbiota has been shown to negatively impact human digestive physiology, to have pathogenic effects on the immune system, and, in turn, cause exaggerated neuroinflammation. Given the tremendous amount of evidence linking neuroinflammation with neural dysfunction, it is no surprise that the Western diet has been implicated in the development of many diseases and disorders of the brain, including memory impairments, neurodegenerative disorders, and depression. In this review, we discuss each of these concepts to understand how what we eat can lead to cognitive and psychiatric diseases.

## 1. Introduction

Over the last several decades, the prevalence of chronic disease and mental illness has increased substantially, and this increase in disease occurrence has coincided with the increased consumption of ultra-processed foods and excessive energy intake [1]. There is growing evidence that this relationship may be more than just coincidental. Researchers investigating gut–brain connections and their implications to human health have found that the bidirectional signaling between the brain and gut is vital for maintaining homeostasis and regulation of the central nervous system (CNS) and enteric nervous system (ENS). Increasing evidence suggests that appropriate diversity of gut microbiota, the collective microorganisms including bacteria, archaea, and fungi that live in the digestive tract, plays an essential role and greatly impacts this communication. The mechanisms behind this communication are largely unknown, and most of the recent studies have focused on understanding how gut microbiota can affect the brain. This review details the impact that the evolution of the human diet has had on the connection from the gut to the brain, with a focus on the role of gut microbes and immune signaling as potential mechanisms. In addition, it summarizes findings linking gut microbiota to psychiatric and neurodegenerative disorders.

## 2. Evolution of the Human Diet

The advent of plant and animal agriculture some 10,000 years ago caused a permanent environmental shift that had a profound effect on human physiology and health [1,2]. The alteration in nutrient characteristics of formally wild food was initially subtle but changed rapidly with technological advances in food processing and preservation following the Industrial Revolution. This shift from consuming preagricultural wild foods, mostly from hunting and gathering, to postindustrial semi-processed and ultra-processed foods less than 200 years ago has significantly impacted the commensal microbial species that inhabit the human gastrointestinal (GI) tract and, therefore, our digestive, immune, and neural physiology [3]. This alteration in human physiology is correlated with an increase in chronic disease prevalence [1]. One likely explanation for the negative impact on human health is the lack of evolutionary experience our physiology has with these modern foods. Strikingly, over 70% of the daily energy consumed by individuals in the United States come from foods that would have contributed to very little or none of the energy consumed by our preagricultural ancestors [4].

Even for foods that have been consumed by both pre- and post-agricultural humans, the macronutrient composition has drastically shifted, especially within the last 100 years. For example, the consumption of animal meats has been altered due to modern food-processing techniques. Available data suggest the fatty acid profile of domesticated, factory-farmed meat is heavily enriched with saturated fatty acids (SFAs) at levels that were not possible prior to animal agriculture [1]. This is due to the elimination of the seasonal depletion of SFAs, and the accompanied increase in monounsaturated (MUFAs) and polyunsaturated fatty acids (PUFAs) that occurs in wild animals [5]. Thus, year-round consumption of SFA-enriched meats was not typical of the early hominid diet. Furthermore, ~99% of all beef consumed in the US today is derived from grain-fed, factory-farmed cattle, which have a higher SFA content and lower content of omega-3 fatty acids (n-3 FAs) and MUFAs, compared to grass-fed cattle [6].

The impact of the elimination of n-3 FAs from the modern Western diet on digestive, immune, and brain health should not be underestimated. A new database of the fossil record suggests a turning point in human evolution parallels the introduction of seafood and, thus, n-3 FAs to the hominid diet [7]. In fact, seafood consumption was a staple of the early modern human diet and constituted up to 50% of the energy consumption [7,8]. This inclusion of n-3 FAs, particularly docosahexaenoic acid (DHA), in the hominid diet likely contributed to the evolution of modern human immune and nervous systems [9]. As such, the recent depletion of n-3 FAs due to Western diet consumption deprives brain and immune cells of nutrients that are essential for their optimal functioning. 

The altered macronutrient composition of animal meats, coupled with the increase of refined carbohydrates and sugars in ultra-processed foods that are abundant in grocery stores today, has become a staple of the standard American, or Western, diet [10]. These foods include cake, cookies, crackers, sugary breakfast cereals, pizza, potato chips, soft drinks, and ice cream and have little to no similarities to the types of foods consumed by our ancestors, such as wild game, nuts, fruits, and berries, during which our digestive, immune, and nervous systems evolved [1]. The rate at which certain nutrients are being consumed in excess by humans of the postindustrial era far outpaces the time necessary for evolutionary adaptation of the digestive system to these foods. Thus, these foods could be considered “pathogenic” in that they elicit an immune response in both peripheral and central nervous system tissues, which in turn can lead to profound effects on mood and cognitive function.

In addition to the evolution of the human gut, immune system, and brain, billions of microbial organisms have also evolved along with humans to make us their optimal host species. To achieve this symbiotic relationship, microbes had to evolve functions that were essential for their host’s survival and this involved a direct relationship with the food the host consumed [10]. The rest of this review will focus on the impact of Western diet consumption on brain health and function via gut–brain connections, focusing on microbiota and immune-mediated mechanisms.

## 3. Gut–Brain Axis

**Figure 1:**
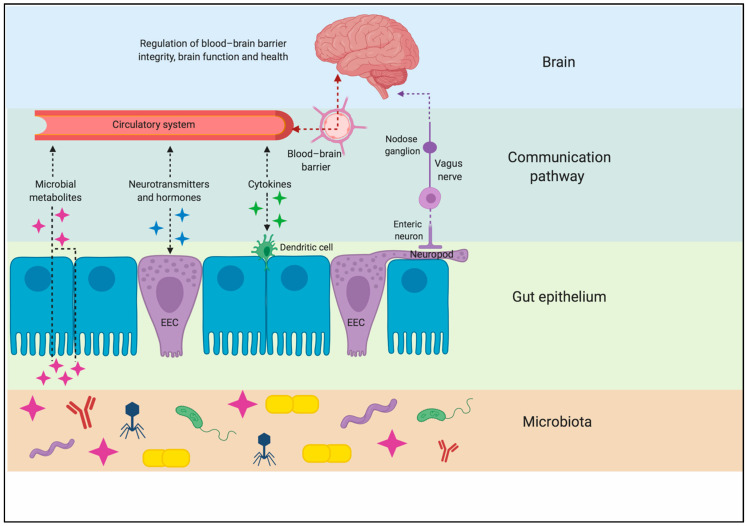
Pathways of communication along the gut–brain axis. A complex interaction of endocrine, immune, and neural cells forms a signaling system that works by sensing changes in microbiota metabolites in the gut and communicating the changes to the brain via both circulatory and neural routes. EEC: enteroendocrine cell. Created with BioRender.com.

While it was once thought that the brain was a privileged organ immune to the dynamic changes occurring in the digestive, immune, and circulatory systems, it is now clear that the food an organism consumes can directly impact brain function. The gut–brain axis is a complex neurohumoral communication network that is imperative for maintaining metabolic homeostasis. This bidirectional system consists of the CNS, ENS, autonomic nervous system (ANS) (including the sympathetic and parasympathetic divisions), neuroendocrine connections, immunological systems, and intestinal microbiota [11,12]. There are three main modes of communication between the gut and the brain: (1) neuronal messages carried by vagal afferents, (2) endocrine messages carried by gut hormones, and (3) immune messages carried by cytokines [12] (Figure 1). The majority of known axial effects on energy homeostasis are a consequence of neural and hormonal gut-derived signals, as the GI tract possesses over 500 million neurons and is capable of producing an array of hormones [13]. Due to the large innervation in the GI tract, ingested components can initiate signals to the CNS regarding macronutrient content and caloric value through individualized, nutrient-specific sensory mechanisms located throughout the GI tract [14].

Gut hormones initiate the majority of signaling and communication within the gut–brain axis in response to pre-absorptive nutrients. These are released by the enteroendocrine cells (EECs), which are located throughout the epithelium of the GI tract, with many having an apical cell membrane covered in microvilli, which are open to, and directly contact, the luminal contents [15]. Digestion and nutrient absorption occur within the stomach and small intestine. Thus, these organs are highly innervated, as they are the primary sites responsible for nutrient-sensing. This dense area of innervation originates from the vagal and splanchnic nerves [16]. Vagal fibers extend into the lamina propria of the intestinal villi, terminate at the basolateral cell membrane of the EECs, and express receptors for gut hormones and peptides, leading to receptor activation and neuronal stimulation [17]. Whereas the ENS controls intestinal function locally via reflex actions, it also plays a role in transmitting nutrient-derived signals to vagal afferents, contributing to the gut–brain axis [18]. Intrinsic ENS neurons are proximally located to EECs and various primary afferent nerve terminals. These neurons are stimulated by intestinal nutrient infusion, and they stimulate vagal afferent fibers in the gut [18]. These studies suggest that the gut–brain neuronal signaling axis is initiated by nutrient-induced gut hormone secretion.

**Figure 2:**
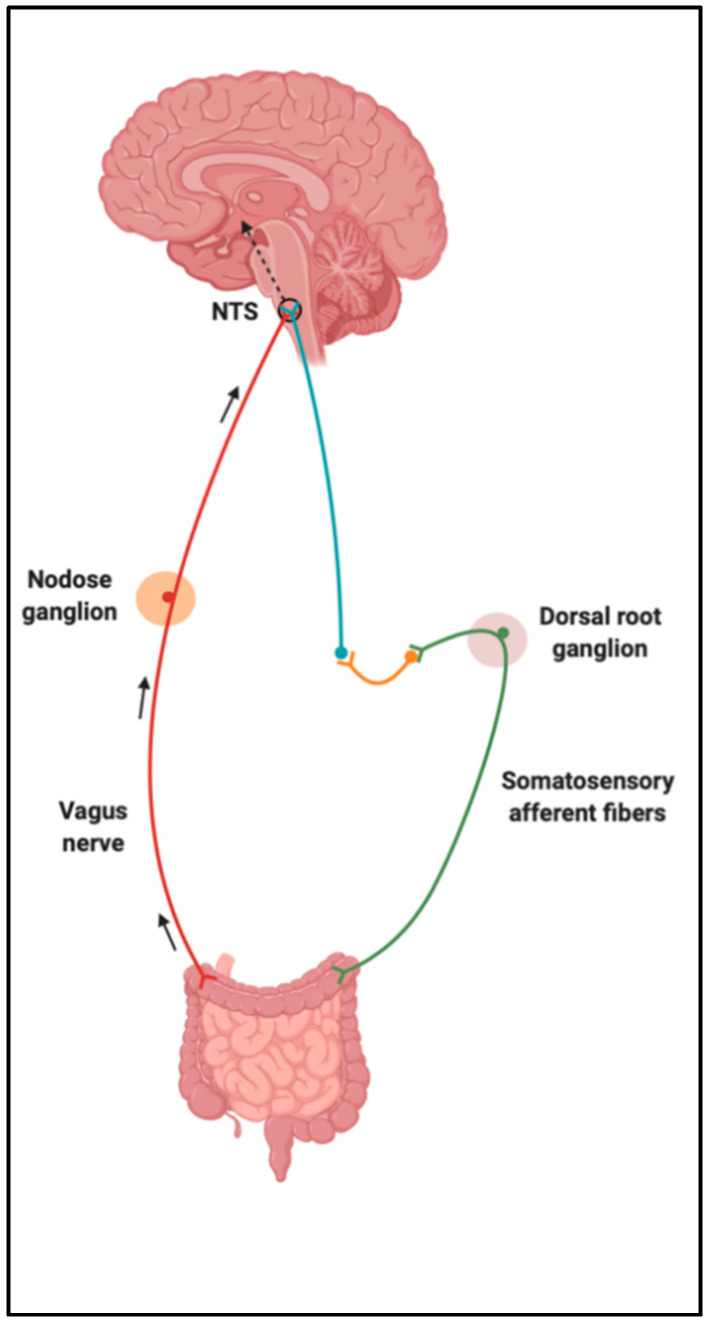
Sensory information is carried from vagus and somatosensory afferent fibers to the nucleus of the solitary tract (NTS). Created with BioRender.com.

Upon food consumption, sensory information is carried from the GI vagal and/or somatosensory (spinal) afferent fibers to the nucleus of the solitary tract (NTS) [19]. Vagal afferents converge in the NTS of the dorsal vagal complex (DVC) within the brainstem, and somatosensory afferents synapse with neurons in the posteromarginal nucleus of the spinal dorsal horn, which then projects to the NTS (Figure 2). NTS neurons integrate and carry these gut-derived signals to several higher-order centers of the brain, such as the melanocortin system in the hypothalamus [20,21]. The hypothalamus performs the function of integrating homeostatic signals from the hindbrain and peripheral humoral signals that transduce information of nutrient consumption and energy expenditure [16]. Some of the hypothalamic regions unified by circuitries to control feeding behaviors and regulate energy homeostasis include the arcuate (ARC), paraventricular, ventromedial and dorsomedial nuclei, and the lateral hypothalamic area [21]. It is clear that nutrient-sensing occurs in the gut and triggers neural and/or humoral pathways, contributing to this bidirectional communication system.

## 4. Gut Microbiota and Its Impact on Brain Function

As mentioned earlier, the human microbiota consists of a diverse and dynamic population of microbes, including bacteria, archaea, viruses, fungi, and protozoa that establish a symbiotic relationship with the host [22]. Modern sequencing technology has identified at least 1000 species and more than 7000 strains of bacteria, estimating that more than 10^14^ bacterial cells populate the GI tract [12,22]. The intestinal microbiota is associated with the integrity of the epithelial barrier, and the maintenance of intestinal metabolic and immune homeostasis. Also, evidence from primarily rodent studies suggests that microbiota may play a direct role in brain function.

The gut microbiota transforms dietary components into metabolites, such as short-chain fatty acids (SCFA) and amino acid derivatives. SCFA are small organic monocarboxylic acids produced by colonic fermentation of dietary fiber and complex plant-based polysaccharides. These have essential metabolic and signaling functions that can modulate blood–brain barrier (BBB) integrity and brain function. SCFAs can access the BBB via the bloodstream to impact its integrity directly [22]. For example, in germ-free mice that were monocolonized with *Clostridium tyrobutyricum,* a bacterium known to primarily produce butyrate, BBB permeability was shown to be decreased by an upregulation of tight junction proteins [23]. After traumatic brain injury, an intravenous administration of sodium butyrate prevented BBB breakdown and promoted neurogenesis [24]. Thus, it is plausible that modulating SCFA levels could be useful in preventing neural dysfunction. In another study, germ-free mice displayed defects in microglia, the resident immune cells of the brain. They exhibited altered cell proportions and an immature phenotype, including more segments, longer processes, and greater numbers of branching and terminal points. They demonstrated that maturation of microglial cells during postnatal development was rescued with SCFAs [25]. Additional work is needed to understand how gut microbiota can be manipulated to achieve an optimum balance of SCFAs in the periphery and the brain to protect cognitive health [22].

Gut microbiota also play an essential role in the catabolism of amino acids, whose products can influence the balance of neurotransmitter production, critical for proper brain functioning [26]. It has been reported that different bacteria can synthesize and release neurotransmitters. Species of the genera *Lactobacillus* and *Bifidobacterium* metabolize glutamate, a free amino acid and excitatory neurotransmitter in the brain, to produce γ- aminobutyric acid (GABA), a major inhibitory neurotransmitter [27]. Studies support the idea that glutamate activates N-methyl-aspartate type of glutamate receptors (NMDARs) in endothelial cells, which leads to excess calcium signaling and downstream nitric oxide production to promote BBB permeability [28,29]. Thus, having the appropriate balance between glutamate and GABA curtails this deleterious effect. The gut microbiota also regulates serotonin by altering levels of its precursors. *Clostridium sporogenes* secretes decarboxylases, which converts tryptophan, an essential amino acid, into tryptamine and is involved in the release of serotonin [30]. Tryptophan from the GI tract can enter the circulation, cross the BBB, and initiate serotonin synthesis in the brain, making tryptophan metabolism in the GI tract critical for central serotonergic signaling [12]. Tryptophan depletion has been demonstrated to affect a variety of cognitive processes, including learning and memory in both healthy and diseased individuals [31]. Taken together, these findings highlight the important role gut microbiota play in neurotransmitter regulation, and in turn, in BBB permeability and neuroprotective functions [22].

## 5. Gut Microbiota in the Aging Population

Aging is a complex process affecting physiological, genomic, metabolic, and immunological functions. It has been defined as a “state of increased vulnerability to poor resolution of homeostasis after a stressor, which increases the risk of adverse outcomes” [32]. Recent work has helped to understand various metabolic-associated mechanisms and hallmarks that underlie the complex processes of age-associated disturbances to the immune system such as inflammation and metabolic dysfunction [33]. Interestingly, these metabolic perturbances are associated in older adults with physical and cognitive declines leading to chronic diseases including obesity, autoimmune diseases, diabetes, and neurodegenerative diseases [33,34]. Considering that gut microbiota are closely associated with pro- and anti-inflammatory balance, as well as immune and gut–brain axis, these old-age related clinical issues could increase vulnerability to disease by causing alterations in the microbiota of older people [35,36].

Gut microbes do not age, but the incidences of comorbidities associated with gut microbiota tend to increase as the host grows older [37,38]. Older individuals have a different gut microbiota profile compared to healthy younger adults, and this difference is associated with lifestyle and dietary schedule, reduced mobility, weakened immune strength, altered gut morphology and physiology, infections, medications, etc. [39]. Studies have found a lower Firmicutes to Bacteroidetes ratio, and reduction in species producing SCFAs, in particular butyrate, in older as compared to younger adults [40,41]. Additionally, levels of opportunists such as enterobacteria *C. perfringens* and *C. difficile* are increased in older adults [39,42]. It should be noted that these aging-associated changes in the gut microbiota may vary according to the geographical location, since different results have been observed in older populations in Europe, for example, where lifestyle attitudes and diet are dramatically different than in the United States [43].

## 6. Western Diet and the Gut–Immune–Brain Axis

Given that the gut microbiota is a complex ecosystem that evolved with the hosts’ digestive, immune, and nervous systems, drastic alterations in the host organism’s diet will undoubtedly have a significant impact on the gut microbiota and, therefore, the overall health of the organism. Thus, the Western diet has presented an extreme challenge to the gut microbiota, potentially due to its lack of evolutionary relevance mentioned earlier. Indeed, the Western diet negatively impacts human digestive physiology and can have pathogenic effects on the immune system, which is likely mediated by the gut microbiota. This leads to changes in the CNS and, ultimately, in behavior [3,10,44].

### 6.1. Fatty Acids

Excessive accumulation of SFAs via consumption of the Western diet can act as a proinflammatory signal in the periphery, as well as in the brain [45,46,47,48]. It has been shown that the SFAs palmitic, lauric, and stearic acid can all independently activate toll-like receptors (TLRs) located on the surface membrane of macrophages located in the gut and in surrounding tissues after nutrient absorption [10,49,50]. Much like the endotoxin lipopolysaccharide (LPS), SFA-induced TLR4 activation promotes the phosphorylation of the Ikappa B alpha (IkBa) protein and subsequent disinhibition of nuclear factor kappa B (NF-kB), ultimately leading to the synthesis and release of proinflammatory cytokines, such as interleukin-1beta (IL-1β), interleukin-6 (IL-6), tumor necrosis factor alpha (TNFα), and interferon gamma (INFγ) [10,50]. While SFAs can directly impact inflammation, they also alter the gut microbiota by raising the ratio of Gram-negative bacteria that produce LPS, which is the natural ligand for TLR4 [51]. Moreover, excessive SFA consumption can increase gut permeability, further promoting the leakage of LPS into the bloodstream and causing inflammation, a state known as metabolic endotoxemia [51].

SFAs can also be transported across the BBB via multiple transport protein-mediated mechanisms, where they bind to TLRs on resident microglia and elicit a proinflammatory response through similar mechanisms described in peripheral macrophages [52,53]. It is worth noting that SFA-induced neuroinflammation occurs rapidly and is thought to precede systemic inflammation [54]. This phenomenon is first localized to the hypothalamus and NTS [55,56], where it can alter neural circuits regulating energy balance and further promote excess nutrient consumption [57,58]. However, chronic SFA consumption in adult rodents, or acute SFA consumption in aged rodents, can lead to inflammation in other regions, such as the hippocampus and amygdala, where it can have deleterious effects on learning and memory and contribute to neurodegenerative pathology [45,56,59,60,61,62].

### 6.2. Refined Carbohydrates and Sugar

Another staple of the Western diet is refined carbohydrates, which are grains that have been processed to remove all bran and fiber [10]. Examples include white bread, white rice, pasta, starch, sucrose, and fructose syrup. In rodents, consumption of these foods alter gut microbes at every phylogenic level, with significant group differences in 25% of gut microbes at the family level [44]. Specifically, sugar consumption in rodents results in an increase in Enterobacteriaceae, which has been associated with both intestinal and brain inflammation [63]. Furthermore, rodents consuming high concentrations of sugar have reduced levels of *Lactobacilli*, which facilitate transport of SCFAs [64]. Refined carbohydrate and sugar consumption also has a direct impact on brain function. Preclinical studies show that excess consumption of these nutrients leads to hyperglycemic conditions in the periphery and the brain, which can directly impact glial and neuronal metabolism and ultimately illicit neuroinflammation and synaptic impairments [65,66,67,68]. This hyperglycemia-induced neuroinflammation impairs learning and memory and increases depressive- and anxiety-like behavior in rodents [66,67,69,70,71]. These behaviors have been causally linked to neuroinflammation, as blockade of inflammation reverses or prevents these behavioral phenotypes [72]. Importantly, similar results to these preclinical findings have also been shown in humans. For examples, hyperglycemia has been causally linked to increases in circulating proinflammatory cytokines in human subjects with and without impaired glucose tolerance [73]. Furthermore, acute increases in glucose were associated with impaired cognitive performance in older individuals with type 2 diabetes, suggesting a link between hyperglycemia and cognition in humans [74].

### 6.3. Fiber

Due to high consumption of refined carbohydrates and simple sugars, the Western diet is deficient in fiber. Fiber is a plant-based nutrient and is resistant to intestinal and pancreatic enzymes that typically aid in the digestion of food [10]. Therefore, humans rely on gut bacteria in the colon to ferment and metabolize the fiber we consume. Gut bacteria use fiber to produce SCFAs, which are absorbed into the bloodstream and can readily diffuse across the BBB [10,44]. Once in the brain, they can bind to receptors located on microglia and produce an anti-inflammatory effect [75]. SCFAs are also integral in regulating peripheral immune function, which can indirectly impact the brain [75]. Due to the combination of fiber deficiency, which results in a decline in the bacteria that produce SCFAs, and the excessive sugar intake, which results in a decline in SCFA-transporting bacteria, Western diet consumption results in a significant decline in immune- and brain-regulating SCFAs.

## 7. Western Diet and Brain Health

As mentioned in the beginning of this review, the emergence of the Western diet is tightly correlated with an increase in chronic disease prevalence, including obesity, diabetes, heart disease, certain cancers, and disorders of the GI tract, such as irritable bowel syndrome (IBS) and inflammatory bowel diseases (IBDs) [10]. However, until recently, the impact of these foods on brain health was often overlooked. Because of the interconnectedness of the digestive, immune, and nervous systems, it is no surprise that the Western diet has been implicated in the development of myriad diseases and disorders of the brain. 

**Figure 3:**
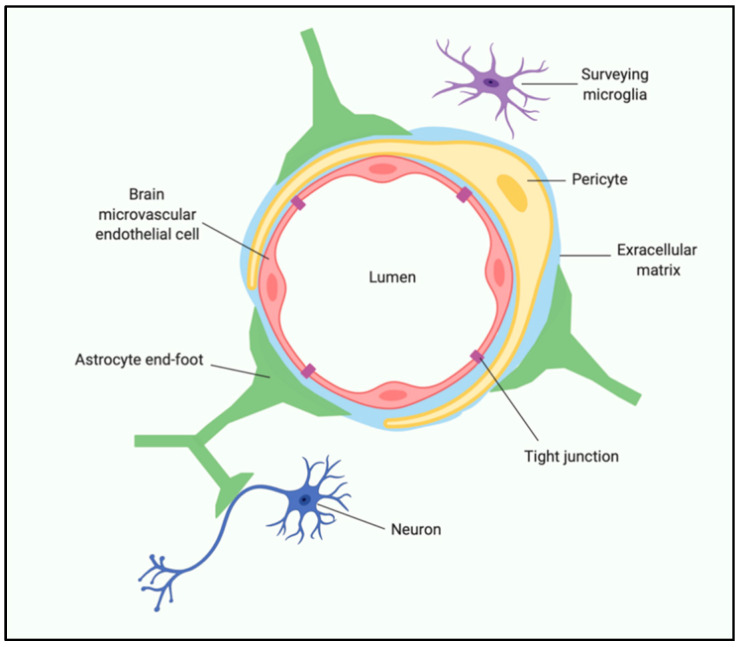
Schematic representation of the human blood–brain barrier. Created with BioRender.com.

One of the predominant mechanisms by which gut dysbiosis (when bacterial colonies in the GI tract are disrupted in a way that is detrimental to the host) and exaggerated peripheral immune activity impacts brain function is by altering the integrity of the BBB. The BBB is a dynamic, highly regulated, specific cellular system comprised of multiple cell types. Brain microvascular endothelial cells (BMEC), pericytes, astrocytes, neurons, microglia, and extracellular matrix (ECM) all contribute to regulating BBB stability and function [22] (Figure 3). Maintaining the BBB integrity is essential for proper synaptic functioning, information processing, and neuronal connectivity. BBB breakdown is known to cause increased permeability, reduction of tight junctions, pericyte detachment, and disruption of basement membrane [76]. The associated pericyte degeneration allows toxic blood-derived molecules, nutrients, cells, and microorganisms to enter the brain, which can initiate pathways of inflammation and neurodegeneration [25]. Accumulation of neurotoxic material and reduced blood flow can activate microglia and astrocytes, causing an inflammatory response, secreting neurotoxic cytokines and chemokines [77]. Also, age-related changes in BBB properties can be observed at an anatomical and physiological level, which includes decreased cortical and white matter microvascular density, decreased capillary lumen size, and reduced number of mitochondria per endothelial cell, suggesting an energy-dependent process [78]. More recent studies in rodents have shown age-related defects in glucose, amino acids, and hormone transport across the BBB, which is associated with cognitive decline [79].

Evidence suggests that when gut microbiota are in a state of dysbiosis, the messages sent to the brain propagate unhealthy signals manifesting in low-grade inflammation, increased oxidative stress, unbalanced energy homeostasis, and a general increase in cellular degeneration [44,80]. Recent studies propose that microbial dysbiosis contributes to the pathology of multiple neurological conditions and diseases, including cognitive impairment, neurodegeneration, and depression [12].

### 7.1. Cognitive Impairment and Aging

Clinical and preclinical data suggest that the consumption of a Western diet high in SFAs can lead to significant deficits in learning and memory [81,82,83,84,85,86]. In adult rodents, this occurs following chronic consumption of a high fat diet (HFD) and appears to be the result of exaggerated neuroinflammation [56,62,87]. Interestingly, short-term (3-day) consumption of a HFD is sufficient to evoke an amplified inflammatory response in the hippocampus and amygdala in aged rats, which leads to a subsequent impairment of hippocampal- and amygdalar-dependent learning and memory, likely by compromising synaptic plasticity [59,60,88,89]. This effect is blocked with an IL-1 receptor antagonist, suggesting the proinflammatory response is critical for the diet-induced memory deficits in aged rats [60]. This age-specific effect of short-term HFD consumption on neuroinflammation and cognition has important implications for the development of neurodegenerative disease and dementias, which are discussed below.

Over the last decade, the connection between Western diet consumption, gut dysbiosis, and cognition have begun to be elucidated in preclinical models. Indeed, Western diet consumption decreases populations of microbes in the phylum Bacteroidetes and increases Firmicutes and Proteobacteria in adult rodents [44]. These shifts in microbiota composition are tightly correlated with cognitive impairments and poor cognitive flexibility [44]. Recently, a functional link between changes in gut microbiota and cognition has been established in mice. Fecal/cecal transplants from adult mice fed a Western diet to adult mice that were pretreated with an antibiotic and fed a control diet resulted in an increase in anxiety-like behavior and impaired contextual fear conditioning in the control mice [90]. These findings suggest a Western diet-altered microbiota alone, independent of an obesity phenotype, is sufficient to disrupt cognitive functioning, though the specific bacterial species responsible were not identified in this study.

### 7.2. Neurodegenerative Disorders

Alzheimer’s disease (AD) is characterized by severe deficits in memory, cognition, and motor functions. The most prominent neurohistopathological characteristics are the aggregation of amyloid-beta (Aβ) peptide plaques and hyperphosphorylated tau tangles [91]. Furthermore, degradations in synaptic neurites and synaptic plasticity are prominent phenomena in the early stages of AD that correlate well with the progressive decline of cognitive functions [92,93,94,95]. Excesses of several bacterial species have been found to produce or aggravate the production of Aβ plaques, including *Bacillus subtilis*, *Escherichia coli*, *Klebsiella pneumoniae*, *Mycobacterium* spp., *Salmonella* spp., *Staphylococcus aureus*, and *Streptococcus* spp. [96]. However, the most consistent alterations in gut microbiota flora observed in AD are the decreased abundance of anti-inflammatory bacteria such as Firmicutes and Bacteroidetes, which can lead to an increase in inflammation levels in the plasma and subsequently in CNS [97]. AD is also associated with local intestinal inflammation, and, in preclinical AD models, intestinal inflammation and intestinal Aβ levels are tightly correlated with the earliest indicators of brain inflammation and Aβ levels [98]. These data suggest intestinal inflammation is present early in disease onset and, thus, may play a critical role in the pathophysiology of AD. As mentioned earlier, exaggerated neuroinflammation leads to synaptic plasticity deficits and abrupt cognitive deficits. These studies demonstrate that microbiota dysbiosis can trigger several pathologies in the gut and brain, such as inflammation, cerebrovascular degeneration, Aβ plaques aggregation, and tau pathology, all of which support the hypothesis that gut microbiota have a strong connection with the pathogenesis of AD.

Parkinson’s disease (PD), the second most common neurodegenerative disorder, is a movement disorder characterized by the degeneration of the dopamine-producing zona compacta neurons of the substantia nigra [99]. Alpha-synuclein (αSyn) protein production and aggregation are the major neuropathologic markers in PD and can be found in the GI tract prior to their detection in the brain [22,99]. This αSyn aggregation is likely directly related to intestinal inflammation, as αSyn is critical for the innate immune response in the gut [98]. Briefly, αSyn accumulation has been observed following bacterial and viral infection in the gut, and αSyn has been implicated as a chemoattractant that enhances the local immune response. Thus, intestinal inflammation has been proposed as an environmental link to neurodegeneration, with disease beginning in the gut and spreading to the brain via the vagus nerve [100]. The sequence of events could involve age-, infection-, or dysbiosis-driven αSyn accumulation in the intestine, leading to the synaptic transmission of pathological forms of αSyn from the ENS to the vagus nerve, and a retrograde axonal transport along the vagus to the brainstem [22]. In one study in which the gut microbiota from PD patients and age-matched controls were quantitatively measured using gas chromatography, significantly reduced concentrations of the bacterial phylum Bacteroidetes and the bacterial family Prevotellaceae were detected in PD patients [101]. *Prevotella* breaks down complex carbohydrates, providing SCFAs, as well as thiamine and folate as byproducts, thereby promoting a healthy intestinal environment. Decreased numbers of *Prevotella* is likely to result in reduced production of these important micronutrients [99,100]. These results suggest that changes in gut microbiota could have a direct effect on neurodegenerative disease via the gut–brain axis.

### 7.3. Depression

The inflammatory hypothesis of depression has gained traction in recent years. Multiple meta analyses have indicated that patients diagnosed with clinical depression have higher levels of circulating proinflammatory markers, such as IL-1β, IL-6, TNFα, and C-reactive protein (CRP), than non-depressed individuals [102,103]. Indeed, there is significant overlap between sickness behaviors and common manifestations of depression symptoms, including fatigue, sleep disturbances, social withdrawal, anhedonia, and loss of appetite [104]. Rodent studies have shown that proinflammatory cytokines can cross the BBB and stimulate the synthesis and release of central proinflammatory cytokines by microglia in brain regions critical for mood regulation and reward processing [104].

As mentioned above, the Western diet can independently promote central inflammation via direct nutrient–microglia interactions and peripheral inflammation via gut–immune interactions [44,53,54]. The combination of increased central inflammation from brain uptake of proinflammatory nutrients, gut dysbiosis, increased BBB permeability, and increased peripheral inflammation caused by Western diet consumption could directly contribute to the development of depressive symptoms. In support of this notion, depression is a common comorbidity associated with GI-related diseases, such as IBS and IBD, and treatment of these conditions can also improve an individual’s brain health [105,106,107]. In preclinical models, diet-induced neuroinflammation is also associated with depressive- and anxiety-like behaviors, adding further credence to the concept that diet and nutrition directly impacts brain health through immune-mediated mechanisms [108,109].

The human gut microbiota has also been implicated in depression [110]. When comparing the gut microbiota of individuals diagnosed with major depressive disorder (MDD) to those of healthy individuals, there is a reported decrease in species richness and diversity in those with MDD [111]. Notably, there are alterations in the abundance of different genera within Bacteroidetes, Firmicutes, Proteobacteria, and Actinobacteria phyla in patients with MDD [112]. This decrease in species richness and diversity is typically associated with increased inflammation [113]. In support of the notion that the gut microbiota may be causally linked to depression in humans, two recent meta-analyses of randomized controlled clinical trials showed that the consumption of probiotics significantly improves depressive symptoms in MDD patients [114,115]. Given that probiotics directly alter gut microbiota composition, this provides compelling evidence that the microbiota is causally linked to mood regulation in humans. Mouse studies, using fecal microbiota transplants (FMTs), have provided compelling support for gut microbiota alterations directly causing changes to the brain and behavior. Briefly, transplantation of fecal microbiota from chronically stressed mice that display depressive-like behavior to recipient mice caused the recipients to have increased depressive-like behaviors and increased neuroinflammation relative to controls [116]. This is corroborated to an extent in humans by the finding that a human-to-mouse FMT from patients with MDD causes depressive-like behavior in recipient mice [117]. Lastly, a case study of a 79-year-old woman with MDD showed that a FMT from a non-depressed donor improved clinical symptoms of depression in this patient [118]. While exciting, further research needs to be conducted on a larger scale to evaluate the safety and efficacy of this practice in human patients.

## 8. Conclusions and Future Directions

**Figure 4:**
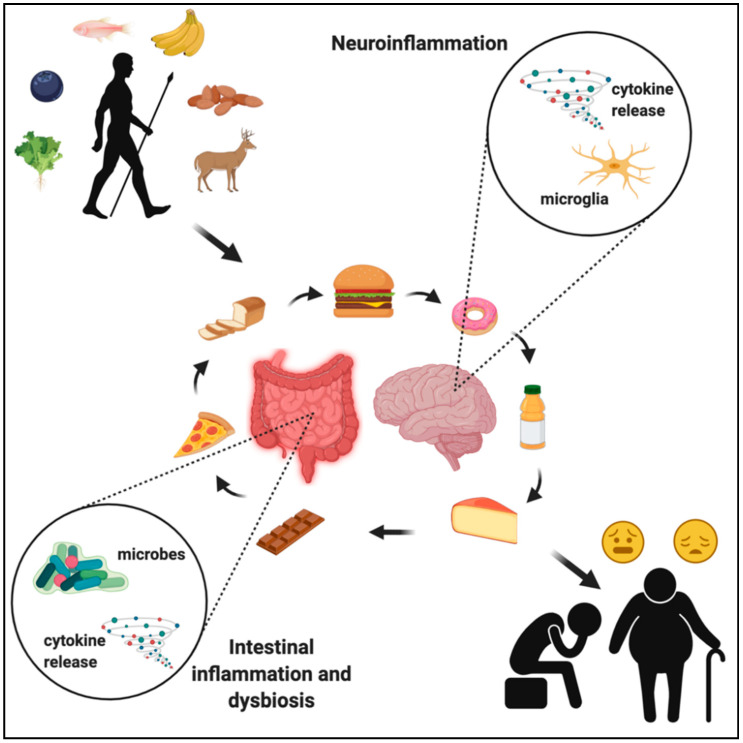
The evolution of the human diet, from natural food sources to ultra-processed foodstuffs, has led to drastic changes in the gut microbiota, which has negatively impacted immune signaling in both the intestines and the brain, contributing to widespread chronic disease in the developed world. Created with BioRender.com.

Knowing the evolutionary history of food consumption, preparation, and nutritional content provides an important perspective with regard to understanding how the food we eat impacts our brain health. It is clear that the human gut microbiota has not had sufficient time to adapt to certain modern foods, thus evoking pathogenic responses by the immune system, leading to harmful neuroinflammation, and causing cognitive and psychiatric disorders (Figure 4). Further investigation of bacterial metabolites and their effects on hormone production, immune signaling, and neural function will help to fully understand brain responses to age- and disease-associated alterations in gut microbiota. Dietary and microbial modulation are promising avenues toward targeting neurodegenerative and neurological diseases, but the current data on specific mechanisms are limited. These limitations are often due to the lack of non-invasive techniques that allow imaging of specific cellular phenotypes in the human brain, thus not allowing for direct quantification of how nutrition and the gut microbiota impacts cellular function in humans. Given that preclinical models suggest neuroimmune mechanisms are driving the link among nutrition, the gut microbiota, and brain function, future studies should focus on improving techniques to evaluate neuroinflammation in humans. For example, further development of imaging methods of gliosis or positron emission tomography (PET) imaging of microglia-specific markers in response to tightly controlled nutritional and/or probiotic interventions is one potential avenue to better understand the impact of nutrition and probiotics on neuroinflammation in humans [57,119].

Conceptually, some additional areas that should be studied are (1) the influence of gut microbiota and the gut–brain axis on repairing and remodeling the CNS as well as on the development and progression of neurodegenerative disease; (2) the transgenomic metabolic interactions among gut microbiota, in order to decipher and learn to optimize gut health; and (3) the development of cost-effective techniques to map an individual’s complete gut microbiota, in order to help physicians create a personalized therapeutic regimen. Advances in metabolomics and metagenomics will provide greater understanding of the potential health benefits of gut microbiota. In the meantime, a diet composed of natural whole foods with minimal processing can help prevent and alleviate some of the burden caused by chronic disease. Such a diet has increased evolutionary relevance to human physiology and, perhaps more importantly, microbial biology compared to the typical ultra-processed and artificial Western diet [110,120]. In addition to our effort to reverse chronic disease states, which has not been met with large-scale success, perhaps we should increase our energy into developing preventative and personalized medicine, an effort in which the role of diet will be at the forefront.

## Data Availability

Data sharing not applicable.
